# An analytical retrospective study to determine the prevalence of childhood obesity and assess the effectiveness of current surveillance

**DOI:** 10.1017/S146342362000033X

**Published:** 2020-08-14

**Authors:** Lucille Mclean, Richard Phillips

**Affiliations:** School of Clinical Medicine, University of Cambridge, Hills road, Cambridge CB20SP, UK

**Keywords:** childhood obesity, obesity, paediatric, primary care

## Abstract

**Background:**

The childhood obesity epidemic is a public health crisis. Most surveillance occurs in primary care, yet there is limited guidance for the detection and management of childhood obesity.

**Aims and methodology:**

We sought to establish the overweight and obesity prevalence in children aged 4–11 years old in a single primary care centre. Furthermore, we assessed whether appropriate weight management referrals were considered and determined the average duration since children last had their height and weight measured.

**Findings:**

We detected overweight or obesity status in 29.0% of our cohort, and only one-third (31.1%) of eligible children had evidence that appropriate weight management referral was considered. The average duration since last height and weight measurement was 20.3 months.

**Discussion:**

Childhood obesity requires an effective and inclusive solution, and in this report, we explore whether increased surveillance is necessary and how we might achieve this.

## Background

Childhood obesity is a national and international public health crisis. In England, nearly one-third of children have overweight or obesity status (Health and Social Care Information Centre, 2019), and the most recent data indicate that the prevalence of obesity in 4- and 5-year-olds is increasing (Health and Social Care Information Centre, 2019). Obesity can cause various health problems both in childhood and in later life, which include cardiovascular disease, type 2 diabetes and musculoskeletal problems, as well as poor mental health and depression (Upadhyay *et al.*, 2018). Timely detection and management of childhood obesity are therefore of the upmost importance.

In 2016, the UK government formally addressed this issue with a Childhood Obesity Plan (Department of Health & Social Care, 2016). This saw the introduction of a soft drinks industry levy, a sugar reduction programme and involved various other recommendations to promote better child health. Whilst these are proactive preventative measures, there remains a significant lack of guidance on how to identify children with overweight or obesity status, and thereafter how best to support them. Recently, there has been debate about whether a UK-wide screening programme for childhood obesity is needed; however, this is not currently recommended by the UK National Screening Committee (UK NSC).

There is some form of screening in England as part of the National Child Measurement Programme (NCMP), which measures the height and weight of eligible children at 4–5 and 10–11 years old (reception and primary school year 6, respectively). In 2018/2019, this showed that the prevalence of childhood obesity more than doubles between these two age groups, from 9.7% in 4- to 5-year-olds to 20.2% in 10- to 11-year-olds (Health and Social Care Information Centre, 2019). Currently, the Royal College of Paediatrics and Child Health (RCPCH) is recommending that the NCMP is to be extended to include children after birth, before school and in adolescence (Royal College of Paediatric and Child Health, 2017). Whilst this will provide invaluable additional information on trends in childhood obesity, it provides no extra surveillance for children between 4–5 and 10–11 years old, where we know that the prevalence of obesity significantly increases. Detection of childhood obesity during this period is predominantly the responsibility of primary care clinicians, partly because an estimated third of parents are unable to recognise when their children are overweight (Black *et al.*, 2015).

Within primary care, however, there are very limited guidelines on when children should have their height and weight measured and when it is appropriate to refer to weight management services. National Institute for Health and Care Excellence (NICE) recommends that clinicians ‘use clinical judgement to decide when to measure a person’s height and weight’ (NICE, 2014), but this is open to interpretation, and clinicians may disagree on when measurements should be performed. Additionally, despite guidance that children with a BMI >91st percentile should be referred to weight management services (Welbourn *et al.*, 2018), many primary care centres are not equipped with the IT systems to calculate paediatric BMI percentiles, thus cannot refer appropriately.

In this study, we aimed to establish the overweight and obesity prevalence in children at a single primary care centre and to assess whether appropriate referrals were considered. Additionally, we aimed to determine the average duration since children last had their height and weight measured and discuss whether more should be done to improve childhood obesity surveillance.

## Methodology

Electronic patient records at a single UK primary care centre, with a registered population of 5694, were searched retrospectively using a single eligibility criterion of primary school-aged children (4–11 years old). Three hundred fifty children were identified but 16 were excluded due to incomplete records. Patient records were searched on 21 January 2020 for the most recent height and weight measurements of each child. These were used to calculate an age- and sex-specific BMI percentile for 334 eligible children using an National Health Service Body Mass Index calculator. BMI percentiles were used to identify children with overweight and obesity status in the cohort, defining overweight as ‘85th–94th BMI percentile’ and obesity as ‘≥95th BMI percentile’, as per the RCPCH (Royal College of Paediatric and Child Health, 2017). Additional analysis of the 10- to 11-year-old subgroup was performed to assess if the prevalence of obesity had increased from their measurements at 4- to 5-year-olds. Of 94 10- to 11-year-olds in the cohort, 65 had sufficient patient records to be included in this analysis.

From the total cohort, a further search was performed to identify children eligible for weight management referral (BMI >91st percentile), as per current NICE guidelines. These children’s records were searched for documented evidence that a referral had been considered.

Finally, a calculation was made to determine when each of the 334 children last had their height and weight measured. This was used to determine the average duration since last measurement.

## Results

In January 2020, 334 children of primary school age (4–11 years old) were identified as registered patients at a single primary care centre. The mean age was 7.9 years, with approximately equal distributions between males (*n* = 160) and females (*n* = 154) (Table 1).

**Table 1.** Entire cohort grouped by age and sex

Age group (years)	No. of males in age group, *n* (%)	No. of females in age group, *n* (%)
4	11 (55)	9 (45)
5	13 (37.1)	22 (62.9)
6	20 (50)	20 (50)
7	30 (56.6)	23 (43.4)
8	22 (56.4)	17 (43.6)
9	29 (54.7)	24 (45.3)
10	22 (45.8)	26 (54.2)
11	24 (52.2)	22 (47.8)
Total	160 (51)	154 (49)

Overweight or obesity status was detected in 29.0% of children included in the cohort (overweight = 10.2%; obesity = 18.9%). In the subgroup analysis of 65 10- to 11-year-olds, the prevalence of obesity had increased almost threefold from 6.2% when they were 4–5 years old to 18.5% at their current age. The current 4- to 5-year-olds in our cohort had a prevalence of obesity of 16.4%.

22% of children were eligible for weight management referral (>91st BMI percentile); however, only one-third (31.1%; *n* = 23) of these had documented evidence that this had been considered. An actual referral to weight management services was only made for one child.

**Figure 1.**
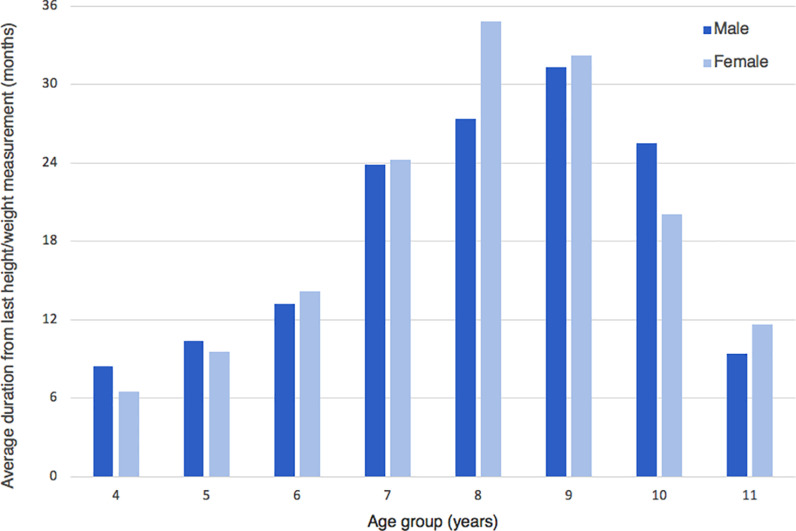
Average duration from last height/weight measurement (in months) grouped by ages 4 to 11

The average duration since children last had their height and weight measured was 20.3 months (range 0.13−70.4), with significant variation between age groups (Figure 1), which likely reflects involvement in the NCMP. Notably, children with asthma (10.2%) had an average duration since last measurement of 10 months (range 0.46−47.6). The greatest difference between children with and without asthma was seen at 9 years old, where children with asthma were on average measured 11.02 months ago compared to 31.74 months for those without asthma.

## Discussion

As part of this study, we aimed to establish the prevalence of childhood obesity at a single primary care centre. We found that 29.0% of 4- to 11-year-olds had overweight or obesity status, which reflects the national statistic of 28% (Health and Social Care Information Centre, 2019). The prevalence of childhood obesity has increased significantly over the last two decades (Van Jaarsveld and Gulliford, 2015), irrespective of various government initiatives. The most recent NCMP report shows that the prevalence of childhood obesity is still increasing, and thus a combination of both prevention and surveillance must be adopted to tackle this epidemic.

In the subgroup analysis, we found that the prevalence of obesity in current 10- to 11-year-olds had increased almost threefold from when they were 4–5 years old. This highlights a critical period in child health where increased surveillance for childhood obesity may be necessary. Early intensive interventions have successfully led to a BMI reduction in preschool children (Foster *et al.*, 2015), and thus, more regular height and weight measurements may be justified. Currently, the UK NSC does not recommend routine weight surveillance in children due to a lack of evidence showing its utility. This has been contested by the RCPCH, who maintain that a lack of evidence does not equate to ‘a lack of effectiveness’ (Royal College of Paediatrics and Child Health, 2018). It is our opinion that whilst there may not be strong evidence for routine weight measurements as part of a screening programme, there is likely a benefit to additional weight surveillance in primary care. Notably, the current 4- to 5-year-olds registered at the practice have an obesity prevalence of 16.4%. If these children follow the same trajectory as their predecessors, then we can predict that up to half of them will develop obesity over the next 6 years.

Most of the current efforts to tackle childhood obesity are in primary prevention. However, we must continue to support children who have become overweight or developed obesity. NICE currently recommends that children with a BMI >91st percentile are to be referred to weight management services (NICE, 2014). However, our study found that only 31.1% of eligible children had documented evidence that a referral had been considered. There is evidence that interventions in this age group can lead to a significant weight reduction (Ho et al., 2012); therefore, further investigation is needed to determine why appropriate referrals are not made. We propose that this shortcoming is multifactorial, with both personal and systematic elements. Firstly, healthcare professionals may lack the confidence to initiate conversations about weight management in children, especially as many parents disagree with feedback that their child is overweight or has obesity (Park *et al.*, 2013). In our study, healthcare professionals considered referrals for 23 children; however, only one child was successfully referred to weight management services. From our observations, some parents can represent a significant barrier to service uptake even where referrals are justified. Additionally, there are systematic barriers such as the IT systems used in primary care. These often cannot easily calculate a paediatric BMI, and this prevents GPs from easily tracking changes in a child’s BMI percentile.

Finally, we also looked at the average duration of time since children last had their height and weight measured. We found that the average duration since last measurement was 20.3 months, with the longest duration being 70.4 months (5.9 years). We found significant variation in duration since last measurement between children of different age groups (Figure 1). Only children aged 4, 5 and 11 years had an average duration since last measurement of less than 12 months, which likely reflects involvement in the NCMP. Nine-year-old children had the highest average duration since last measurement at almost 32 months (2.6 years). Currently, the RCPCH proposes extending the NCMP to include children at birth, before school and in adolescence. We support this recommendation but also propose that the NCMP is extended to include children at 7–8 years old, in between the two current measurements. This would provide an invaluable opportunity to identify children who have recently become overweight or developed obesity, so that early interventions can be used to alter their trajectory. Furthermore, we believe that height and weight measurements in children should be financially incentivised via the quality of framework system, as is seen in adults. This may encourage primary care clinicians to include height and weight measurements as part of their paediatric consultations.

We wish to acknowledge that our cohort was taken from a single primary care centre that had a smaller proportion of children than the national average. It would be interesting to complete the same study at a practice with a higher proportion of children to see how the results may differ.

## Conclusion

Childhood obesity requires an effective and inclusive solution. We have highlighted a critical period in child health where children may benefit from additional surveillance of their height and weight. We have also shown that current surveillance may not be sufficient and have made suggestions for how this may be improved.
